# Apple (*Malus domestica* Borkh.) Cultivar ‘Majda’, a Naturally Non-Browning Cultivar: An Assessment of Its Qualities

**DOI:** 10.3390/plants10071402

**Published:** 2021-07-09

**Authors:** Anka Cebulj, Andreja Vanzo, Joze Hladnik, Damijana Kastelec, Urska Vrhovsek

**Affiliations:** 1 Agricultural Institute of Slovenia, Department of Fruit Growing, Viticulture and Oenology, Hacquetova ulica 17, 1000 Ljubljana, Slovenia; andreja.vanzo@kis.si (A.V.); joze.hladnik@kis.si (J.H.); 2 Biotechnical Faculty, University of Ljubljana, Jamnikarjeva 101, 1000 Ljubljana, Slovenia; damijana.kastelec@bf.uni-lj.si; 3 Research and Innovation Centre, Fondazione Edmund Mach, Via E. Mach 1, 38010 San Michele all’Adige, Italy; urska.vrhovsek@fmach.it

**Keywords:** non-browning, polyphenol oxidase, phenolics, vitamin C, glutathione

## Abstract

Browning of apple and apple products has been a topic of numerous research and there is a great number of methods available for browning prevention. However, one of the most efficient ways, and the one most acceptable for the consumers, is the selection of a non-browning cultivar. Cultivar ‘Majda’ is a Slovenian cultivar, a cross between ‘Jonatan’ and ‘Golden Noble’. In this study, it was thoroughly examined and compared to the well-known cultivar ‘Golden Delicious’ with the aim to decipher the reason for non-browning. We have determined the content of sugars, organic acids, vitamin C, glutathione and phenolics in apple flesh, with the addition of phenolic content in apple peel and leaves. The change in color in halves and pomace was also measured and the activity of peroxidase (POX) and polyphenol oxidase (PPO) were determined. Additionally, the analyses of flesh were repeated post-storage. The most prominent results were high acidity (malic acid), low phenol content, especially hydroxycinnamic acid and flavan-3-ol content of cultivar ‘Majda’ in comparison to ‘Golden Delicious’, and no difference in PPO activity between cultivars. After the overview of the results, we believe that both low phenol content and high reduced glutathione content impact the non-browning characteristics of cultivar ‘Majda’.

## 1. Introduction

Browning is associated with deterioration. Its prevention, either by using additives or choosing resistant cultivars, has taken a great part in horticultural and food research. The conventional method to inhibit browning in fruit has been to utilize sulfites [1]. However, due to health concerns, alternative means of controlling enzymatic browning are required [2]. In addition to numerous food processing techniques for prevention of browning, the initial decision on cultivar selection is crucial for all further steps. We now have several known cultivars with a smaller rate or lack of browning, such as arctic apples, ‘Ambrosia’, ‘Eden’, ‘Aori27’, etc. [3,4,5]. The mechanism behind browning is oxidation of polyphenols. Oxidation occurs when tissues are damaged, either by improper handling causing bruising or by processing, cutting, peeling or grinding. Due to damaged cells, the phenolics come into contact with polyphenol oxidase (PPO). In intact cells, PPO seems to have little activity towards phenolics [6]. In addition to the phenolic content, the activity of PPO is the reason for the development of browning [7]. PPO interacts with phenolic substrates and molecular oxygen, since it is a bi-metalloenzyme with two copper-binding domains [8]. The primary reaction is initiated by PPO accumulated in plastids, and not by *de novo* formed enzyme, although high activation occurs in time after a cell-damaging event. Furthermore, phenolic concentration increases in time after wounding [9]. What are the main mechanisms behind non-browning cultivars? Arctic apple cultivars were genetically engineered with a transgene that produces specific RNAs to silence PPO genes [5]. Cultivar ‘Eden’ has a low phenolic content [3]. In addition to the low phenolic content, a low PPO activity is also thought to be behind a lack of browning in cultivar ‘Aori 27’ [4]. The lack of browning for cultivar ‘Ambrosia’ is explained by the lower activity of PPO enzyme [10].

Another important quality of a cultivar and its part of defense metabolism are two major low-molecular-weight antioxidants, ascorbic acid (Vitamin C) and glutathione (GSH) [11,12]. Ascorbic acid has an ability to reduce quinones back to phenolic compounds prior to their subsequent reaction to form pigments. While GSH is directly linked to cellular ascorbic acid metabolism through the ascorbate-glutathione cycle, GSH is used as a source of reducing power for the enzymatic regeneration of oxidized ascorbic acid [13]. Furthermore, glutathionyl-chlorogenic acid conjugate was reported in apple juice [14]. Glutathionyl conjugates of hydroxycinnamic acids are known for limiting the browning of grape juice, where GSH interferes by trapping the caftaric acid quinones produced by oxidation in the form of 2-s-glutathionylcaftaric acid [15]. In addition, GSH has a role in biosynthetic pathways, detoxification, antioxidant biochemistry, and redox homeostasis [16].

All the above-mentioned metabolites have different preserving abilities during storage that depend firstly on cultivar and pre- and post-harvest parameters. According to Awad and Jager [17], total phenolics are relatively stable during storage. A good stability of the main antioxidants (including GSH and ascorbic acid) was also reported [18]. Davey and Keulemans [19] reported on the increased GSH content after 3 months of cold storage of several apple cultivars, as well as increased vitamin C content. The increase, and in some cases the decrease, of GSH and vitamin C mainly depended on the cultivar. However, a weak correlation to the harvest time was implicated, as well.

**Figure 1:**
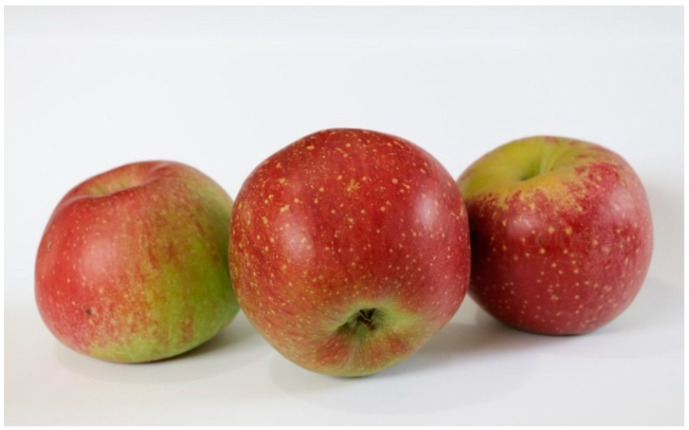
‘Majda’ apples.

Cultivar ‘Majda’ was confirmed as a variety in 1986 and was made from the cross of ‘Jonatan’ and ‘Golden Noble’ [20]. The apple has a dark green basic color with a dark red top color (Figure 1). Even though its non-browning characteristics were described when it was introduced, it is not a well-known or a widely used cultivar. Only a few growers have cv. ‘Majda’ planted in orchards. One of the reasons is probably the color of fruit, which is not as appealing as the color of modern apple cultivars. It has high acidity and is therefore mainly known for its use in processing. Cultivar ‘Golden Delicious’ is a well-known cultivar and in numerous countries, the time of harvest and basic characteristic of cultivars are compared to this cultivar. With respect to this, cultivar ‘Golden Delicious’ was chosen as a comparison to cultivar ‘Majda’. The first phenolic analysis of cultivar ‘Majda’ were made by Persic et al. [21], where they compared several cultivars in terms of phenolic content and browning. They have correlated a stronger oxidation to the high total phenolic content in apples. Their results urged us to focus on this cultivar, to further explore the phenomenon of non-browning of cultivar ‘Majda’.

The aim of this study was to establish the main parameters of inner quality of cultivar ‘Majda’ in comparison to ‘Golden Delicious’. We have determined the content of sugars, organic acids, vitamin C, glutathione (GSH and GSSG), its precursors cysteine, methionine, and phenolics in apple flesh, with the addition of phenolic content in apple peel. Additionally, to establish if the low phenolic content also reflects in leaves, the content of phenolics in leaves was determined. Furthermore, we wanted to decipher the main reasons for its low susceptibility to browning. The change in color in halves and pomace was also measured and the activity of peroxidase and polyphenol oxidase were determined. Moreover, we have repeated the analysis on flesh post-storage to determine if the trait persists after storage.

## 2. Results

### 2.1. Sugars

**Figure 2:**
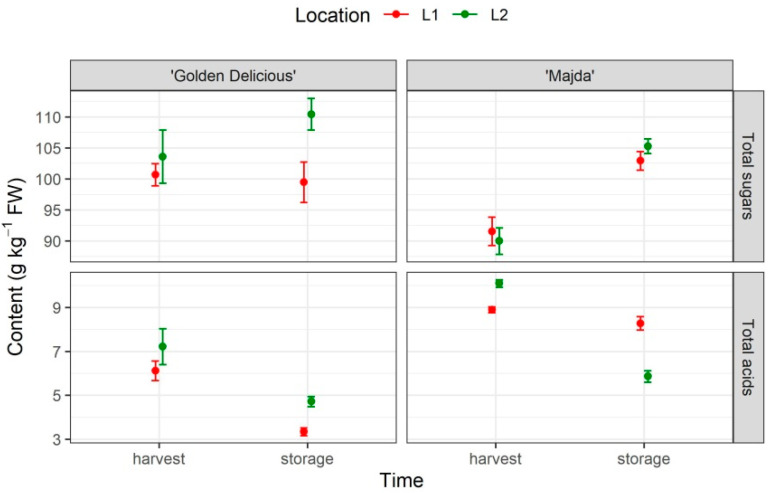
Content of total sugars (g kg^−1^ FW; mean ± SE) and total organic acids (g kg^−1^ FW; mean ± SE) of cultivars ‘Golden Delicious’ and ‘Majda’ at two locations (L1 and L2) at harvest and following storage.

The results of total sugars and organic acids are presented in Figure 2 and their statistical analysis in Table 1. All three factors (cultivar, time, and location) have a statistically significant influence on sugar content. Moreover, the interactions between the cultivar and time as well as the cultivar and location are significant. The contrast analysis showed that there are statistically significant differences in the mean total sugar content between ‘Golden Delicious’ and ‘Majda’ at L2, whereas there were no statistically significant differences between cultivars at L1. However, when also looking at confidence interval, one can see that it is also close to a statistically significant difference at location L1 (Table S1). Following storage, there are statistically significant differences between L1 and L2 with the ‘Golden Delicious’ cultivar. Time had a statistically significant influence on the content of total sugars in ‘Majda’ at both locations. The composition of individual sugars also differs between cultivars (Figure S1), with ‘Majda’ having a higher content of sucrose and sorbitol and a lower content of glucose and fructose.

### 2.2. Organic Acids

Malic and citric acid were quantified among organic acids (Table S2). Fumaric acid was also determined, but its amount was exceptionally low and thereby not quantified. Malic acid is the prevalent acid in apples, which is why its content has a decisive influence on the total acid content in apple. When analyzing all the factors, we can see that there is a statistically significant interaction between all of them. After the contrast analysis was made, statistically significant differences were confirmed between cultivars within locations at harvest and at location L1 following storage. Significant differences in the total acid content following storage in ‘Golden Delicious’ at both locations were noted and in cv. ‘Majda’ at location L2.

### 2.3. pH

**Figure 3:**
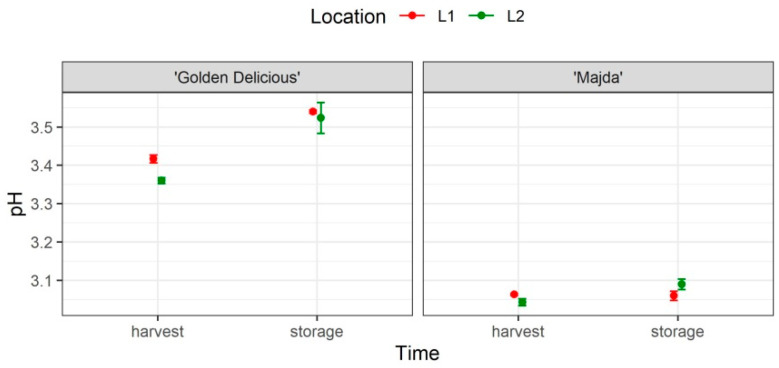
Content of apple juice pH (mean ± SE) of cultivars ‘Golden Delicious’ and ‘Majda’ at two locations (L1 and L2) at harvest and following storage.

The results for apple juice mean that the pH values (±SE) are presented in Figure 3 and statistical analysis in Table 1. The cultivar and time have a statistically significant influence on pH as well as their interaction. Apple juice pH is not influenced by location. The analysis of contrasts (Table S3) confirmed statistically significant differences between cultivars.

### 2.4. Color Change

**Figure 4:**
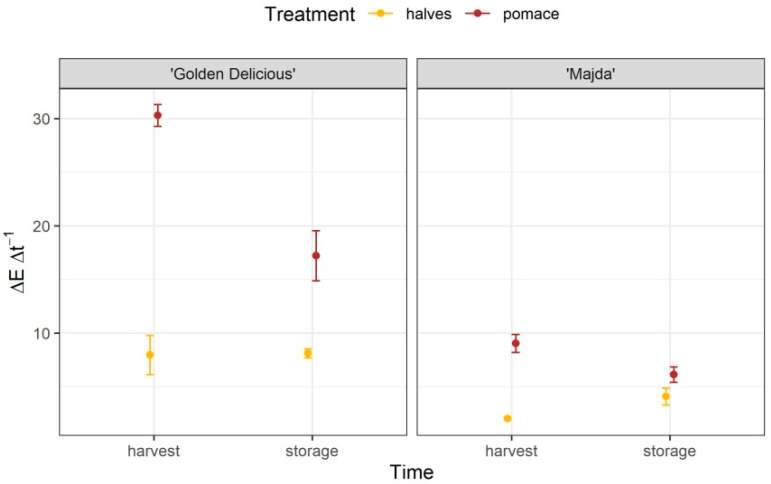
Change in color (ΔE Δt^−1^; mean ± SE) of apple halves after 1 h and pomace after 10 min for cultivars ‘Golden Delicious’ and ‘Majda’ at harvest and following storage.

The change in color is notably different between cultivars (Figure 4 and Table 1). Halves and pomace of cv. ‘Majda’ changed color far less in comparison to ‘Golden Delicious’. The difference was more pronounced after harvest, but even following storage, cv. ‘Majda’ kept its potential of a lower rate of color change. The contrast analysis confirmed statistical differences between cultivars (Table S5). Interestingly, following storage, there were no statistically significant differences between halves and pomace of cv. ‘Majda’.

### 2.5. Vitamin C, Methionine, L-cysteine, GSH and GSSG

**Figure 5:**
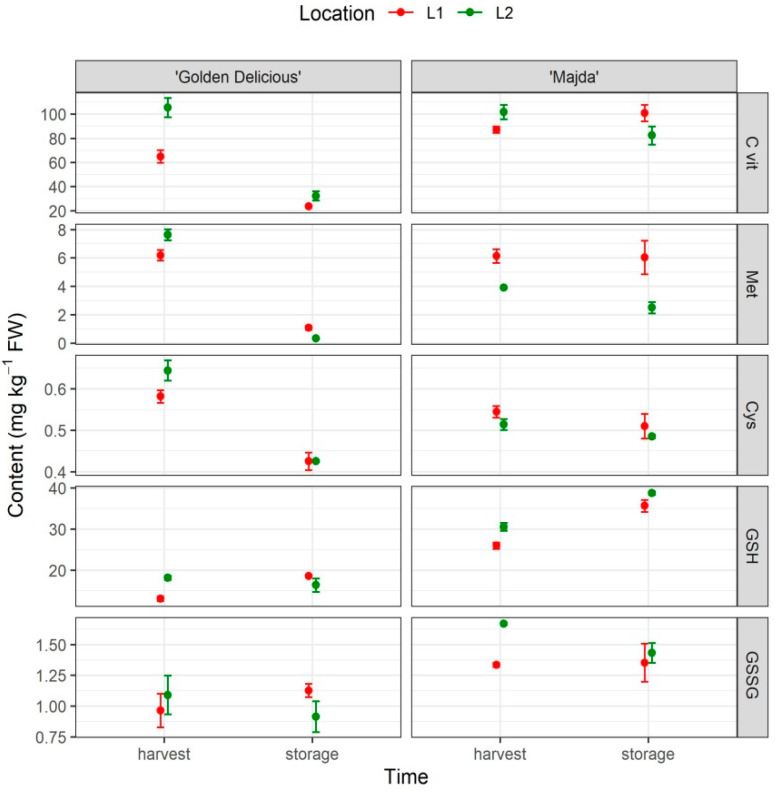
Vitamin C (C vit), methionine (Met), cysteine (Cys), reduced glutathione (GSH), and oxidised glutathione (GSSG) content in apple flesh (mg kg^−1^ FW; mean ± SE) for cultivars ‘Golden Delicious’ and ‘Majda’ at two different locations (L1 and L2) at harvest and following storage.

The content of two major antioxidants in plants, vitamin C and glutathione (GSH and GSSG) as well as GSH precursors L-cysteine and methionine, presented in Figure 5 and their statistics in Table 1. All the three factors (cultivar, time, and location) have a statistically significant influence on the vitamin C content as well as on the methionine and GSH content. The most interesting statistical difference for our study is the vitamin C content in apple flesh following storage, where cv. ‘Majda’ flesh contains a higher content of vitamin C in comparison to cv. ‘Golden Delicious’ (Table S5). In addition, interestingly, there were no statistically significant changes in the vitamin C content in cv. ‘Majda’ between harvest and storage. The same pattern is also visible with methionine and GSH. With the latter content differed already at harvest (between cultivars within locations). Cysteine was highly influenced by the time and interaction between time and cultivar, its content being statistically lower in cv. ‘Golden Delicious’ after storage in comparison to harvest at both locations. The GSSG content depended on the cultivar, the content of GSSG in cv. ‘Golden Delicious’ flesh representing 69% of the content of GSSG in cv. ‘Majda’ flesh. Differences are also represented with the heatmap (Figure S2).

### 2.6. Phenolic Content

**Figure 6:**
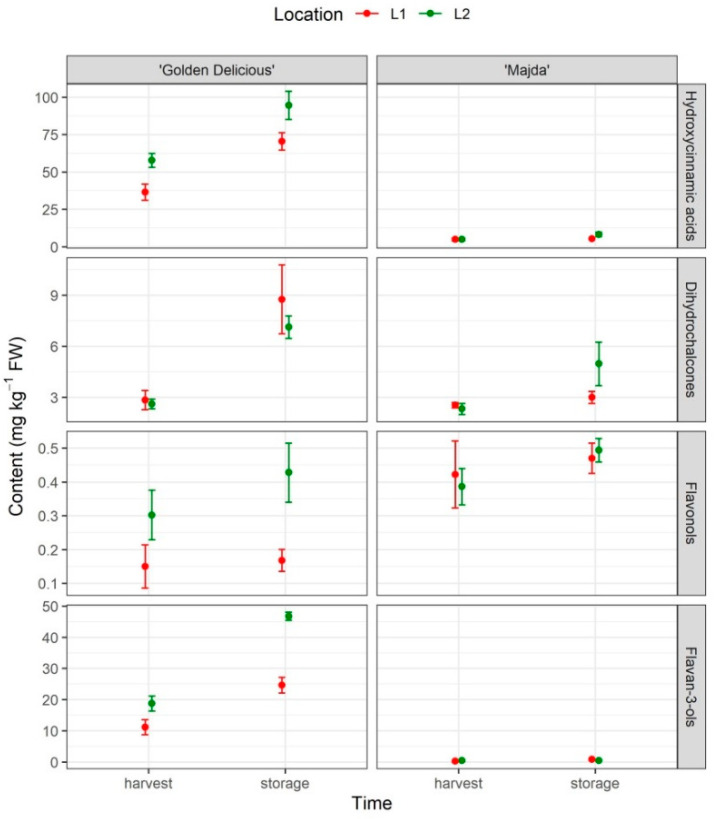
Phenolic content in apple flesh presented in four groups: Hydroxycinnamic acids, dihydrochalcones, flavonols, and flavan-3-ols (mg kg^−1^ FW; mean ± SE) for cultivars ‘Golden Delicious’ and ‘Majda’ at two different locations (L1 and L2) at harvest and following storage.

**Figure 7:**
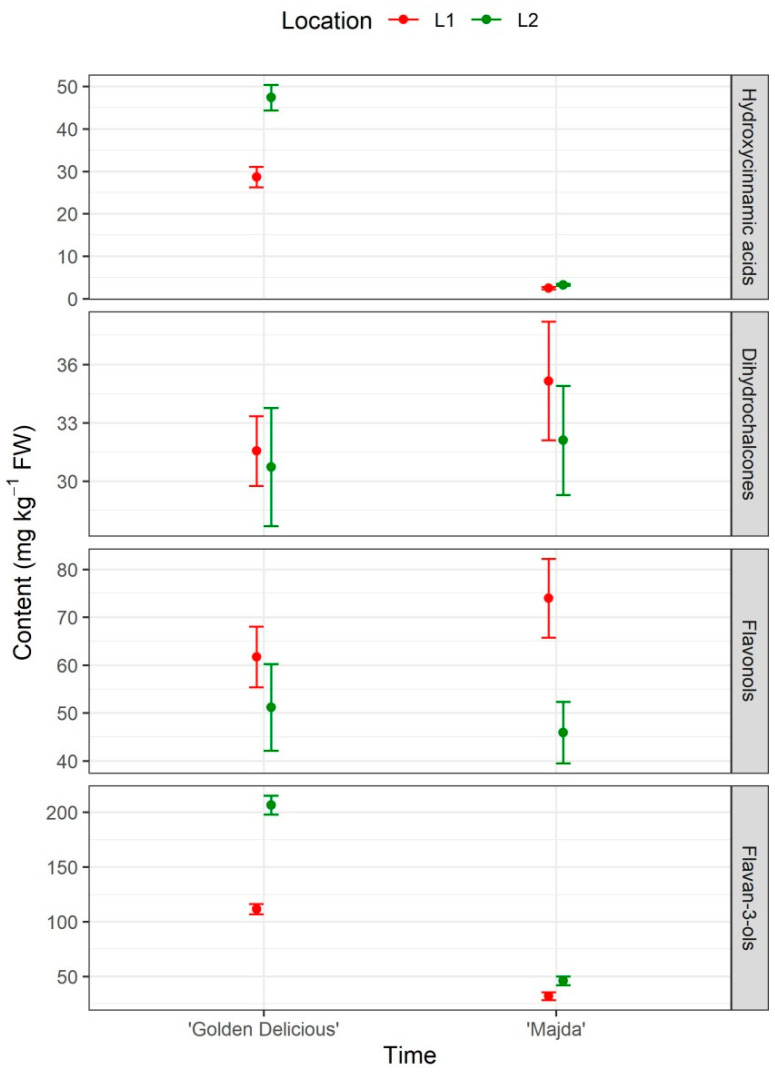
Phenolic content in apple peel presented in four groups: Hydroxycinnamic acids, dihydrochalcones, flavonols, and flavan-3-ols (mg kg^−1^ FW; mean ±SE) for cultivars ‘Golden Delicious’ and ‘Majda’ at two different locations (L1 and L2).

**Figure 8:**
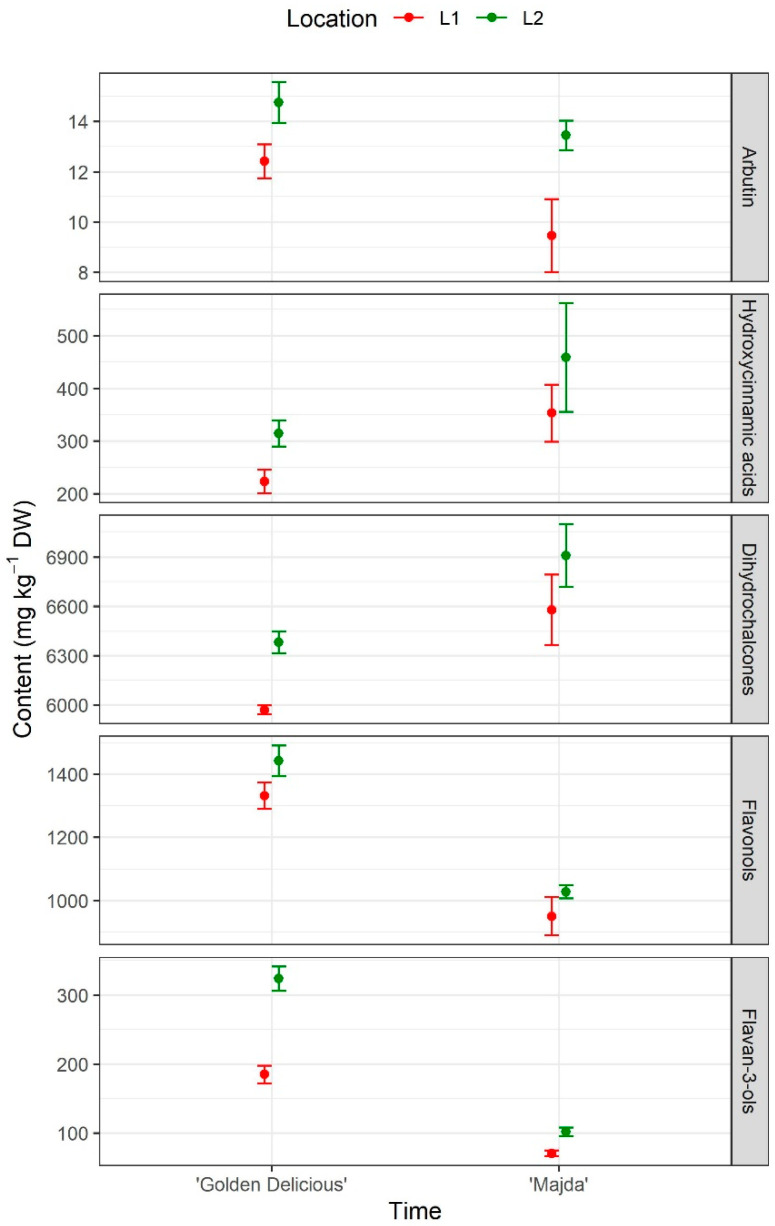
Phenolic content in apple leaves presented as arbutin and four groups: Hydroxycinnamic acids, dihydrochalcones, flavonols, and flavan-3-ols (mg kg^−1^ DW) for cultivars ‘Golden Delicious’ and ‘Majda’ at two different locations (L1 and L2).

Phenolic content was determined in flesh (Figure 6), peel (Figure 7), and apple leaves (Figure 8). Determined phenolics were arranged into four groups (for leaves, five) in which they are presented in Figures and mean contents of individual phenols are presented in Table S6 and Figure S3. Hydroxycinnamic acids: Cryptochlorogenic acid, chlorogenic acid, p-coumaric acid, and neochlorogenic acid; dihydrochalcones: Phloridzin, phloretin 2’-O- xylosyl-glucoside, 3-hydroxyphloridzin, and 3-hydroxyphloretin; flavonols: Quercetin-3-rhamnoside, quercetin-3-rutinoside, and quercetin-3-glycoside + quercetin-3-galactoside; and flavan-3-ols: Catechin, epicatechin, procyanidin B1, procyanidin B2 + B4. In the peel of cv. ‘Majda’, cyanidin-3-galactoside was also determined, but was not included in the results. The 3-hydroxyphloretin was not determined in peel and the phenols neochlorogenic acid, 3-hydroxyphloridzin, 3-hydroxyphloretin, and quercetin-3-rutinoside were not determined in apple flesh. There is a pronounced difference in the phenolic content between cultivars (Table 1). The strongest difference is visible in hydroxycinnamic acid and flavan-3-ol content. The hydroxycinnamic group of phenolics is influenced by all three factors, but there are no interactions between them. Cultivar ‘Golden Delicious’ has on average 11 times higher hydroxycinnamic acid content in comparison to ‘Majda’ (Table S8). At L1, the average content of hydroxycinnamic acids represents 75% content of hydroxycinnamic acids at location L2. On the contrary, the flavan-3-ol content was influenced only by factor cultivar, but with high significance. The dihydrochalcone content differed between cultivars only following storage but not at harvest. However, it was also statistically significantly different within both cultivars comparing apple flesh at harvest and following storage (Table S7). Flavonols, on the other hand, were not influenced by time, but by location in addition to the cultivar. At L1, the contrast analysis confirmed the statistical difference between cultivars.

The pattern is quite similar in apple peel, but the differences are not that extreme (Figure 7 and Table 2). Here, the highest difference is in the content of hydroxycinnamic acids. Compared to cv. ‘Golden Delicious’, cv. ‘Majda’ has about 13 times lower content of hydroxycinnamic acids (Table S8). Location 1 represents 67% content of hydroxycinnamic acids at L2. There were no statistical differences between cultivars in dihydrochalcone and flavonol content, but the latter did vary between locations. Again, flavan-3-ol content differed in cv. ‘Golden Delicious’ (four times higher content) in comparison to cv. ‘Majda’.

In apple leaves, the differences are not as drastic as in fruits but are still present (Figure 8 and Table 2). All the phenolics were influenced by both cultivar and location, with hydroxycinnamic acids and flavonols having weak, statistically significant differences regarding location. However, there were no interactions between factors cultivar and location. Cultivar ‘Golden Delicious’ had twice as much arbutin, 1.4 times higher flavonol content and 2.9 times higher flavan-3-ol content as cv. ‘Majda’ (Table S8). On the other hand, cv. ‘Golden Delicious’ had 70% and 92% content of the cv. ‘Majda’ content of hydroxycinnamic acids and dihydrochalcones, respectively.

### 2.7. PPO and POX

**Figure 9:**
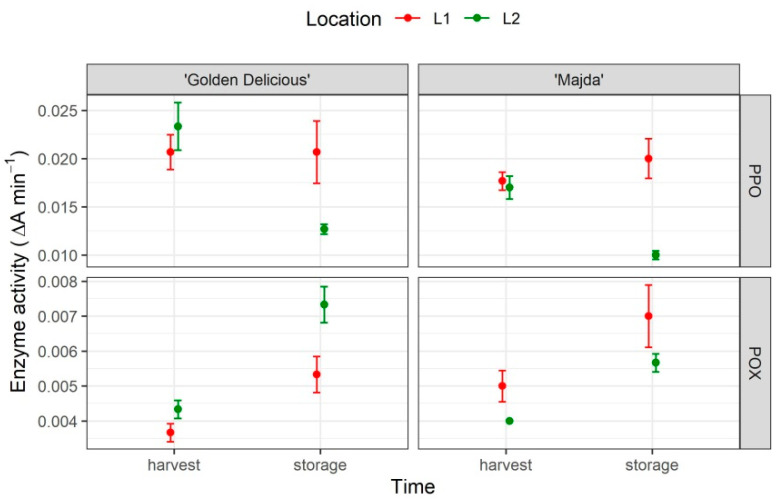
Activity of PPO and POX (ΔA min^−1^) in apple flesh of cultivars ‘Golden Delicious’ and ‘Majda’ at harvest and following storage.

The PPO and POX activities are presented in Figure 9 and statistics in Table 1. There were statistically significant differences in the PPO activity in all three factors. However, the contrast analysis showed no statistically significant differences between cultivars (Table S9). There was a difference in the enzyme activity between apple flesh at harvest and following storage. With POX, time was the only statistically different factor and cultivar:location was the statistically significant interaction.

## 3. Discussion

The results show some promising differences between cultivars. Cultivar ‘Majda’ contains a lower sugar content than cv. ‘Golden Delicious’. Cultivars also differ in individual sugar composition. Even though according to Aprea et al. [22] sorbitol correlates to sweetness the most, its low share in total sugar composition and lower fructose and glucose content in cv. ‘Majda’ do not contribute so much to the sweetness. In addition, the study by Rymenants et al. [23] reported that the perceived sweetness was greatly influenced by the acidity and vice versa. Apples with low acidity are perceived to be sweeter, whereas apples with a strong acidic taste are perceived as less sweet. This is most likely behind the cv. ‘Majda’ taste perception, further supported by Aprea et al. [22], who assessed a negative correlation between malic acid and perceived sweetness (r = −0.449). The higher malic acid content in cv. ‘Majda’ could also be connected to non-browning. In the study by Morimoto et al. [24], they reported on the connection between high acidity alleles and the alleles for non-browning. The major locus controlling apple fruit acidity has been designated as Ma (malic acid), where Ma corresponds to the dominant high acidity or the low pH allele and ma is the low acidity or the high pH allele [25]. For cv. ‘Majda’, it was also believed that the high acidity and low pH are the main factors for its lack of browning. However, in accordance with the previously mentioned data, it might just be the consequence of heritability rather than the key reason for non-browning.

The difference in color change between cultivars is evident. Similar results were also reported by Joshi et al. [26] for cultivar ‘Eden’. In our research, the halves and pomace of cv. ‘Majda’ both had minimal change in color in comparison to cv. ‘Golden Delicious’. Next, we will discuss the main possible causes for this trait.

Both vitamin C and GSH are major antioxidants and this characteristic is also recognized and used in the food industry [2]. However, according to Nicolas [27] the endogenous vitamin C has a marginal role in the prevention of browning. On the contrary, Joshi et al. [26] did find the correlation between the vitamin C content and the whiteness index. In our study, the cultivars did not differ in vitamin C content at harvest, but they did differ in retaining of vitamin C following storage. Following storage, the content of vitamin C remained higher in the cv. ‘Majda’ fruit. Davey and Keulemans [19] reported that cultivars differ substantially in their ability to maintain vitamin C during storage. In their study, the ‘Golden Delicious’ vitamin C decreased following storage, whereas in a few other cultivars it increased. Furthermore, they report on the correlation between fruit vitamin C contents and the harvest date, such that cultivars with the highest vitamin C contents were harvested latest in the season and the lowest contents were found among the early cultivars. It is suggested that this is linked to the cultivar’s capacity for longer storage. However, at this point, we must emphasize on the better storage ability of cultivar ‘Golden Delicious’ in comparison to that of cultivar ‘Majda’ in CA storage (non-published data and experience from growers). The contrast analysis also showed the difference in vitamin C content in the flesh of cv. ‘Golden Delicious’ between locations, whereas the content of vitamin C of cv. ‘Majda’ apples did not vary between locations. It is known that the content of vitamin C is highly influenced by temperature and radiation [28], so this might add an explanation to the higher vitamin C content at L2, but then again, since cv. ‘Majda’ did not show the similar response, this is just a speculation that needs to be addressed in future studies. What we can see, though, is also a higher content of the reduced form of GSH in cv. ‘Golden Delicious’ at harvest at L2. Therefore, location did influence these two antioxidants in cv. ‘Golden Delicious’. However, what stands out with GSH is the statistically significant higher content of GSH in cv. ‘Majda’ at both locations and at both times. What is more, if we look at the phenolic content in flesh, we can significantly see a lower content of hydroxycinnamic acids. In a review on the role of glutathione in winemaking, Kritzinger et al. [29] reported on an interesting observation, the ratio of hydroxycinnamic acid and GSH (HCA/GSH) represents a good indication of the grape must susceptibility to oxidation. A ratio of 0.9–2.2 characterizes a lightly colored must, while the medium and dark must are characterized by 1.1–3.6 and 3.8–5.9 HCA/GSH ratio, respectively. If we compare the HCA/GSH ratio in our samples we can see a great difference, 2.7–3.2 and 0.2–0.3, for cv. ‘Golden Delicious’ and cv. ‘Majda’, respectively. Therefore, we have two aspects we need to take into account when discussing the lack of oxidation in cv. ‘Majda’: Low hydroxycinnamic acid content and high GSH content.

In addition to the low hydroxycinnamic acid, the flavan-3-ols content shows the significant differences in the content of main phenols responsible for browning, namely chlorogenic acid, catechin, and epicatechin [30,31]. Furthermore, at harvest, none of the procyanidins were determined nor were the cryptochlorogenic acid or p-coumaroylquinic acid from flavanols and hydroxycinnamic acids groups, respectively. As Khanizadeh et al. [3] described, the lack of substrate for PPO enzyme may be the cause of non-browning. This statement may be further supported by the results of PPO activity, where no statistically significant differences between our tested cultivars were determined. Following storage, the activity of PPO decreased in both cultivars. The low PPO activity is the main mechanism behind cv. ‘Ambrosia’ non-browning, but based on these results, it is rather the lack of substrate than the lower PPO activity that is the cause, therefore it is more similar to the ‘Eden’ cultivar’s background. The content of dihydrochalcones differed between cultivars only following storage and the flavonol content varied between cultivars merely at L1. Both groups are known for their antioxidant role in apple [32,33], but the content of both groups in apple flesh is quite low. The POX activity was mostly influenced by time, and only the interaction between cultivar and location is statistically significant. With its various activities in plants, it is hard to make any assumptions without any further investigation. Nonetheless, the main differences between cultivars are well summarized by the heatmaps (Figures S1–S3).

Since the high phenolic content is preferred due to its beneficial role in human health [34], we also wanted to evaluate the apple peel from both cultivars. As done in flesh, the low sum of hydroxycinnamic acids and flavan-3-ols also stands out in the peel in cv. ‘Majda’. There were no statistical differences in the contents of dihydrochalcones and flavonols between cultivars. Therefore, the peel has similar properties to flesh. This is not the best in the context of high antioxidant content. However, cv. ‘Majda’ contains anthocyanin cyanidin-3-O-galactoside. Anthocyanins are known for their beneficial role in human health [35].

With interesting results from flesh and peel, we wanted to broaden the knowledge on the phenolic content in cv. ‘Majda’, to see if this trait is present through the whole tree or if it is present just in the fruit. The group of flavonols and flavan-3-ols shows a similar pattern as flesh and peel, while the hydroxycinnamic acids and dihydrochalcones are even higher in cv. ‘Majda’ leaves in comparison to leaves of cv. ‘Golden Delicious’. Both hydroxycinnamic acids and dihydrochalcones are known for their role as antioxidants and the role against various pathogens [32,36]. This might explain the overall lower sensitivity to pathogens of cv. ‘Majda’, which is an important factor for growers. This is encouraging, since in addition to the great fruit attributes, the sensitivity to diseases is a key concern for growers. Location also had an influence on the phenolic content, which was expected due to the different weather conditions.

## 4. Materials and Methods

### 4.1. Plant Material

Leaves and apples were harvested in 2019, at two different locations, in the experimental orchard Brdo pri Lukovici (L1: Continental Slovenia; 46°10’04.8” N, 14°40’55.2” E, altitude 368 m) and in the north-west part of Slovenia, in Sadovnjak Resje (L2: Latitude 46°20” N, longitude 14°12” E, altitude 500 m). Soils on location L1 are Dystric cambisol on fine sediments (clay and silt) with pH = 4.8 (0.01 M CaCl_2_) and organic carbon content 2.1% (Corg) in top 30 cm. On location L2, soils are deep (Eutric cambisol) with a similar organic carbon content (Corg = 2.4%) but not so acidic (pH= 5.8) top soil. Weather conditions on both locations during the vegetation period in 2019 are presented in Table S10 and Figure S4. The amount of precipitation was very similar on both locations (731 mm on L1 and 753 mm on L2). The average daily air temperatures were 16.3 °C on L1 and 17.1 °C on L2, the average daily minimum air temperatures were 10.8 and 11.5 °C and the average daily maximum air temperatures were 21.8 and 23.5 °C, respectively. Rootstock M9 and the slender spindle training system were used in both orchards. In addition to cv. ‘Majda’, cv. ‘Golden Delicious’ was collected as a comparison due to its position in the European market. The leaves were incorporated in the study, to include another angle in comparison. They were sampled on 22 July 2019 at both locations. The leaves were ground in liquid nitrogen and lyophilized. The apples were harvested in their technological ripeness, as determined by firmness, the total soluble solids and starch test of cv. ‘Golden Delicious’ and cv. ‘Majda’ were harvested on 19 and 29 of September 2019, respectively. Both locations were harvested at the same time. Only apples of correct size (1st quality class) were collected and chosen for further handling and analysis. Approximately 30 kg of apples for each location and each cultivar were collected, half of which was immediately placed in cold storage and the rest were peeled, cut directly into liquid nitrogen and stored for the analysis of sugar, organic acid, phenolic content, and enzyme activity (POX and PPO). Apples were stored for 2 months in a cold storage with the temperature of 4 °C.

### 4.2. Color Analysis

Apples were either cut in half or ground with a commercial juicer (Sana juicer by Omega EUJ—707), and the change in color (Spectrophotometer CM-700d; Konica Minolta) was monitored over time, 1 h for the halves and 10 min for the pomace. Parameters a*, b*, and L* were recorded and parameter ΔE was calculated according to the following formula:

$$\Delta E=\sqrt{\Delta a^{2}+\Delta b^{2}+\Delta L^{2}}$$

### 4.3. Sugar and Organic Acid Extraction and Analysis

Frozen flesh was briefly chopped, and 5 g were placed in a 100 mL beaker and then homogenized with 25 mL of twice distilled water using Ultra-Turrax T-25 (Ika-Labortechnik, Staufen im Breisgau, Germany). After 30 min of shaking and 10 min of centrifuge (8400 rpm), the samples were filtered through a 45 µm cellulose filter (Chromafil^®^) into vials until further analysis.

Sugars and organic acids were measured using the Agilent 1100 Series. The Rezex RCM-monosaccharide column (300 × 7.8 mm; Phenomenex, Torrance, CA, USA) for sugars and sorbitol and Rezex ROA organic acid column (300 × 7.8 mm; Phenomenex, Torrance, CA, USA) for organic acids were used for analysis. To determine the sugar content, a method described by Mikulic-Petkovsek et al. [37] was used, with an adjustment of the column temperature to 80 °C. For organic acids, method OIV-MA-AS313-04: R2009 from the Compendium of international methods of analysis was used. The concentrations of sugars and organic acids were calculated with the help of corresponding external standards and were expressed in g kg^−1^ of fresh weight (FW).

### 4.4. Vitamin C Extraction and Analysis

Apples were processed on the day of the harvest to determine their vitamin C content. The method used for the determination is compliant with standard SIST EN 14130:2003 (determination of vitamin C using HPLC (Agilent Technologies, Santa Clara, CA, USA). Apples were quickly peeled, chopped, and immersed in liquid nitrogen prior to being ground in a mortar, using ceramic knife and mortar. Twenty grams of flesh powder were extracted with the metaphosphoric acid (Merck) and placed on the shaker for 30 min. The further procedure was as described in the standard. HPLC (Agilent 1200) with the UV-VIS detector at 265 nm was used for detection. The Gemini C18 110A (250 × 4.6 mm, 5 µm) column with the flow rate 0.7 mL min^−1^ was used. The results are in mg per kg of fresh sample.

### 4.5. Phenolic Content Extraction and Analysis

The phenolic content was determined from the apple powder and leaf powder gained from grounding in the mortar with the help of liquid nitrogen. The extraction and determination of phenolics was made following the protocol of Vrhovsek et al. [38]. Two grams of apple powder were extracted using aqueous 80% methanol (Honeywell, LC-MS Chromasolv). For leaves, a slight modification was made, 1 g of lyophilized leaf powder was used and extracted with 4.8 mL of MeOH:H_2_O (2:1) and 3.2 mL of chloroform (Honeywell, LC-MS Chromasolv). An ultraperformance liquid chromatography-tandem mass spectrometry (UPLC-MS/MS) was used for the analysis. Two µL of samples were injected and processed through 100 × 2.1 mm, 1.8 µm column (Acquitiy HSS T3, Waters), maintained at 40 °C, with the flow rate of 0.4 mL min^−1^. The gradient profile of mobile phases was as described in Vrhovsek et al. [38]. The mass spectrometry detection was performed on Waters Xevo TQMS (Milford, MA, USA), equipped with an electrospray (ESI) source in positive and negative modes [38]. Results are presented in mg kg^−1^ of fresh weight (FW) for flesh and mg kg^−1^ of dry weight for leaves.

### 4.6. Glutathione (GSH, GSSH), Cystein and Methionine Extraction, and Analysis

The frozen apple flesh (−80 °C storage) was ground under cryogenic conditions (−196 °C) using AK11 mill (IKA), rapidly weighted into ice cold MeOH with sample weight to solvent volume (*w*/*v*) ratios of 1:4, shaken at room temperature for 15 min, and centrifuged (10,000× *g*, 4 °C) for 10 min. The volume was adjusted, samples were filtered through a 0.2 µm PVDF filter (Agilent technologies) into dark vials and directly injected onto UHPLC-MS/MS (Agilent technologies), and analyzed as described [39].

### 4.7. POX and PPO Activity Determination

For the enzyme activity, the frozen material from −80 °C of storage was used. We were following the protocol described in Zupan et al. [40] for the preparation of samples for the POX activity and Cebulj et al. [41] for the preparation of samples for the PPO activity. Measurements were made according to the Worthington manual (Worthington Biochemical Corporation, 1972). The enzyme activity was calculated in ΔA min^−1^.

### 4.8. Statistics

A three-factor analysis of variance (ANOVA) with factors cultivar, location, and time was used for the flesh samples. All the factors had two levels: Cultivar (‘Majda’ and ‘Golden Delicious’), location (L1 and L2), and time (harvest and storage). For peel and leaves, the two-factor ANOVA was used (factors cultivar and location). For the determination of sugars, organic acids, and phenolic compounds, the samples were prepared in five replicates. For leaves, four replicates were used. For the enzyme activity, three replicates were performed per treatment (each with three technical repetitions). Some variables were log-transformed before the statistical analysis to meet the assumption of constant variance between treatments. When the ANOVA showed a statistical significance, a contrast analysis was performed with user-defined contrasts. When the interactions between the factors did not show a statistically significant difference, Tukey’s HSD test was used for comparison. The contrasts with statistical significances, heatmap of individual sugars, as well as the means with SE of individual phenolics are presented in supplementary materials (Tables S1–S9 and Figures S1–S3). If the *p*-value for differences between the means was less than 0.05, it was considered statistically significant.

## 5. Conclusions

In conclusion, cv. ‘Majda’ is one of the rare cultivars from traditional cross-breeding with a non-oxidation trait. We have screened some of the main actors of oxidation in apple flesh glutathione, vitamin C, phenolic content, and PPO activity. Based on the results, we conclude that the low phenolic content and high reduced glutathione content are the major reasons for the cv. ‘Majda’ lack of oxidation. We have upgraded the knowledge on cv. ‘Majda’ by also analyzing glutathione, and this feature will have to be further researched. Its high acidity is feasibly a consequence of heredity, rather than the main reason for non-oxidation, but one must not disregard that the high acidity with a low pH can contribute to a slower oxidation by reducing PPO efficiency. This will be further researched by the gene expression analysis. This work also touches upon another topic: Fruit processing. Today, the consumers seek products for a quick consumption, such as prepared snacks, but with as little as possible additives to preserve them. Cultivar ‘Majda’ is suitable for this role and has a great potential for use as a minimally processed product, as well as a regular apple product with no additives for the prevention of oxidation. This will also be addressed in our future research. Furthermore, cultivar ‘Majda’ is not an overly sensitive cultivar and is thereby undemanding for fruit growers.

## Figures and Tables

**Table 1:** Three-factor ANOVA for cultivar (Cul: ‘Golden Delicious’ and ‘Majda’), location (Loc: L1 and L2), and time (T: harvest and storage), as well as their interactions (*p* < 0.05).

	Cul	Loc	T	Cul:Loc	T:Cul	T:Loc	T:Cul:Loc
Total sugars	**	.	***	NS	**	.	NS
Total acids	***	NS	***	**	NS	**	***
pH	***	NS	***	NS	**	NS	NS
Vitamin C	***	**	***	**	***	*	NS
Methionine	***	***	***	NS	***	***	*
Cysteine	NS	NS	***	NS	***	NS	NS
GSH	***	*	***	NS	.	**	*
GSSG	**	NS	NS	NS	NS	NS	NS
Hydroxycinnamic acids	***	**	***	NS	NS	NS	NS
Dihydrochalcones	***	NS	***	NS	**	NS	NS
Flavonols	***	**	.	*	NS	NS	NS
Flavan-3-ols	***	NS	NS	NS	NS	NS	NS
PPO	*	**	**	NS	NS	**	NS
POX	NS	NS	***	**	NS	NS	NS
	**Cul**	**T**	**Trt**	**Cul:T**	**Trt:Cul**	**Trt:T**	**Tr:Cul:T**
ΔE Δt^−1^	***	NS	***	.	NS	***	NS

., Statistically significant differences at *p* < 0.1; *, statistically significant differences at *p* < 0.05; **, statistically significant differences at *p* < 0.01; ***, statistically significant differences at *p* < 0.001; NS: Not significant.

**Table 2:** Two-factor ANOVA for cultivar (Cul: ‘Golden Delicious’ and ‘Majda’) and location (Loc: L1 and L2) and their interactions (*p* < 0.05).

	Cul	Loc	Cul:Loc
**Peel**			
Hydroxycinnamic acids	***	***	NS
Dihydrochalcones	NS	NS	NS
Flavonols	NS	*	NS
Flavan-3-ols	***	***	NS
**Leaves**			
Arbutin	*	**	NS
Hydroxycinnamic acids	*	.	NS
Dihydrochalcones	***	*	NS
Flavonols	***	.	NS
Flavan-3-ols	***	***	NS

., statistically significant differences at *p* < 0.1; *, statistically significant differences at *p* < 0.05; **, statistically significant differences at *p* < 0.01; ***, statistically significant differences at *p* < 0.001; NS: Not significant.

## Data Availability

No additional data is available.

## Supplementary Materials

The following are available online at https://www.mdpi.com/article/10.3390/plants10071402/s1. Figure S1: Heatmap for standardized variables: Individual sugars: Sucrose, Suc; glucose, Glu; fructose, Fru; sorbitol, Sorb; organic acids: Citric acid, Cit_ac; malic acid, Mal_ac; and pH for cultivars ‘Golden Delicious’ (GD) and ‘Majda’ (MA) at two different locations (L1 and L2) at harvest and following storage, dendrogram based on Ward’s clustering squared Euclidian distance. Figure S2: Heatmap for standardized variables: Vitamin C, C vit; methionine, Met; cysteine, Cys; reduced glutathione, GSH; oxidised glutathione, GSSG; polyphenol oxidase, PPO; peroxidase, POX for cultivars ‘Golden Delicious’ (GD) and ‘Majda’ (MA) at two different locations (L1 and L2) at harvest and following storage, dendrogram based on Ward’s clustering squared Euclidian distance. Figure S3: Heatmap for standardized variables: Hydroxycinnamic acids: Cryptochlorogenic acid, X4-CQA; chlorogenic acid, X3-CQA; neochlorogenic acid, X5-CQA; and p-coumaric acid, p_coum_ac; dihydrochalcones: Phloridzin, Phl; phloretin 2’-O- xylosyl-glucoside, Phl_xy_phl; 3-hydroxyphloridzin, X3_Hy_phl; and 3-hydroxyphloretin, X3_Hyd_phl; flavonols: Quercetin-3-rhamnoside, Q-Rha; quercetin-3-rutinoside, Q-Rut; and quercetin-3-glycoside + quercetin-3-galactoside, Q-Glc_Gal; flavan-3-ols: Catechin, Cat; epicatechin, Epicat; procyanidin B1, P_B1; procyanidin B2 + B4, P_B2_B4 for cultivars ‘Golden Delicious’ (GD) and ‘Majda’ (MA) at two different locations (L1 and L2) at harvest and following storage, dendrogram based on Ward’s clustering squared Euclidian distance. Figure S4: Daily precipitations, daily minimum and maximum air temperature for the locations L1 and L2. Table S1: The contrast analysis for total sugars with estimated differences between averages and corresponding 95% confidence intervals (lwr, lower boundary; upr, upper boundary), for time (harvest, storage), location (L1 and L2), and cultivar (GD, ‘Golden Delicious’; MA, ‘Majda’). Table S2: The contrast analysis for total organic acids with estimated differences between averages and corresponding 95% confidence intervals (lwr, lower boundary; upr, upper boundary), for time (harvest, storage), location (L1 and L2), and cultivar (GD, ‘Golden Delicious’; MA, ‘Majda’). Table S3: The contrast analysis for pH with estimated differences between averages and corresponding 95% confidence intervals (lwr, lower boundary; upr, upper boundary), for time (harvest, storage), location (L1 and L2), and cultivar (GD, ‘Golden Delicious’; MA, ‘Majda’). Table S4: The contrast analysis for change in color (ΔE Δt^−1^) with estimated ratios of averages and corresponding 95% confidence intervals (lwr, lower boundary; upr, upper boundary), for time (harvest, storage), location (L1 and L2), and cultivar (GD, ‘Golden Delicious’; MA, ‘Majda’). Table S5: The contrast analysis for vitamin C, methionine, cysteine, and glutathione (GSH and GSSG) with estimated ratios of averages and corresponding 95% confidence intervals (lwr, lower boundary; upr, upper boundary), for time (harvest, storage), location (L1 and L2), and cultivar (GD, ‘Golden Delicious’; MA, ‘Majda’). Table S6: Individual phenols (mean ± SE) in flesh (mg kg^−1^ FW), peels (mg kg^−1^ FW), and leaves (mg kg^−1^ DW). Hydroxycinnamic acids: Cryptochlorogenic acid, 4-CQA; chlorogenic acid, 3-CQA; neochlorogenic acid, 5-CQA; and p-coumaric acid, p_coum_ac; dihydrochalcones: Phloridzin, Phl; phloretin 2’-O- xylosyl-glucoside, Phl_xy_phl; 3-hydroxyphloridzin, 3_Hy_phl; and 3-hydroxyphloretin, 3_Hyd_phl; flavonols: Quercetin-3-rhamnoside, Q-Rha; quercetin-3-rutinoside, Q-Rut; and quercetin-3-glycoside + quercetin-3-galactoside, Q-Glc_Gal; flavan-3-ols: Catechin, Cat; epicatechin, Epicat; procyanidin B1, P_B1; and procyanidin B2 + B4, P_B2_B4. Table S7: The contrast analysis for phenolic groups dihydrochalcones and flavonols in apple flesh with estimated ratios of averages and corresponding 95% confidence intervals (lwr, lower boundary; upr, upper boundary), for time (harvest, storage), location (L1 and L2), and cultivar (GD, ‘Golden Delicious’; MA, ‘Majda’). Table S8: HSD test for phenolic groups, hydroxycinnamic acids, dihydrochalcones, flavonols and flavan-3-ols in apple flesh, peel and leaves with estimated ratios of averages and corresponding 95% confidence intervals (lwr, lower boundary; upr, upper boundary), for time (harvest, storage), location (L1 and L2), and cultivar (GD, ‘Golden Delicious’; MA, ‘Majda’). Table S9: The contrast analysis for PPO and POX with estimated ratios of averages and corresponding 95% confidence intervals (lwr, lower boundary; upr, upper boundary), for time (harvest, storage), location (L1 and L2), and cultivar (GD, ‘Golden Delicious’; MA, ‘Majda’). Table S10: Meteorological conditions during the vegetation period (1. 4. 2019–30. 9. 2021) on locations L1 and L2.

## References

[1] Le Tien C., Vachon C., Mateescu M.-A., Lacroix M. (2001). Milk protein coatings prevent oxidative browning of apples and potatoes. J. Food Sci..

[2] Gacche R.N., Warangkar S.C., Ghole V.S. (2004). Glutathione and cinnamic acid: Natural dietary components used in preventing the process of browning by inhibition of polyphenol oxidase in apple juice. J. Enzym. Inhib. Med. Chem..

[3] Khanizadeh S., Groleau Y., Levasseur A., Charles M.T., Tsao R., Yang R., DeEll J., Hampson C., Toivonen P.T. (2006). SJCA38R6A74 (Eden). HortScience.

[4] Tazawa J., Oshino H., Kon T., Kasai S., Kudo T., Hatsuyama Y. (2019). Genetic characterization of flesh browning trait in apple using the non-browning cultivar ‘Aori 27’. Tree Genet. Genomes.

[5] Waltz E. (2015). Nonbrowning GM apple cleared for market. Nat. Biotechnol..

[6] Mesquita V.L.V., Queiroz C., Eskin N.A.M., Shahidi F. (2013). Enzymatic browning. Biochemistry of Foods.

[7] Amiot M.J., Tacchini M., Aubert S., Nicolas J. (1992). Phenolic composition and browning susceptibility of various apple cultivars at maturity. J. Food Sci..

[8] Demeke T., Morris C.F. (2002). Molecular characterization of wheat polyphenol oxidase (PPO). Theor. Appl. Genet..

[9] Di Guardo M., Tadiello A., Farneti B., Lorenz G., Masuero D., Vrhovsek U., Costa G., Velasco R., Costa F. (2013). A multidisciplinary approach providing new insight into fruit flesh browning physiology in apple (Malus × domestica Borkh). PLoS ONE.

[10] Ambrosia T.M. Why Do Apples Turn Brown?. https://ambrosiaapples.ca/apples-turn-brown/.

[11] Davey M.W., Montagu M.V., Inzé D., Sanmartin M., Kanellis A., Smirnoff N., Benzie I.J., Strain J.J., Favell D., Fletcher J. (2000). Plant L-ascorbic acid: Chemistry, function, metabolism, bioavailability and effects of processing. J. Sci. Food Agric..

[12] May M.J., Vernoux T., Leaver C., Van Montagu M., Inzé D. (1998). Glutathione homeostasis in plants: Implications for environmental sensing and plant development. J. Experiment. Bot..

[13] Noctor G., Foyer C.H. (1998). Ascorbate and glutathione: Keeping active oxygen under control. Annu. Rev. Plant Biol..

[14] McDougall G.J., Foito A., Dobson G., Austin C., Sungurtas J., Su S., Wang L., Feng C., Li S., Wang L. (2020). Glutathionyl-S-chlorogenic acid is present in fruit of Vaccinium species, potato tubers and apple juice. Food Chem..

[15] Singleton V.L., Salgues M., Zaya J., Trousdale E. (1985). Caftaric acid disappearance and conversion to products of enzymic oxidation in grape must and wine. Am. J. Enol. Vitic..

[16] Noctor G., Mhamdi A., Chaouch S., Han Y., Neukermans J., Marquez-Garcia B., Qeval G., Foyer C.H. (2012). Glutathione in plants: An integrated overview. Plant Cell Environ..

[17] Awad M.A., Jager A. (2003). Influences of air and controlled atmosphere storage on the concentration of potentially helathful phenolics in apples and other fruits. Postharvest Biol. Technol..

[18] Li L., Li X., Ban Z., Jiang Y. (2014). Variation in antioxidant metabolites and enzymes of ‘Red Fuji’ apple pulp and peel during cold storage. Int. J. Food Prop..

[19] Davey M.W., Keulemans J. (2004). Determining the potential to breeed for enhanced antioxidant status in *Malus*:mean inter- and intravarietal fruit vitamin C and glutathiones contents at harvest and their evolution during storage. J. Agric. Food Chem..

[20] Crnko J., Marn M. (1986). ‘Majda’ nova Jugoslovanska jablanova sorta za dvojno uporabo. Zbornik Biotehniske Fakultete Univerze Edvarda Kardelja v Ljubljani: Kmetijstvo.

[21] Persic M., Mikulic-Petkovsek M., Slatnar A., Veberic R. (2017). Chemical composition of apple fruit, juice and pomace and the correlation between phenolic content, enzymatic activity and browning. LWT Food Sci. Technol..

[22] Aprea E., Charles M., Endrizzi I., Laura Corollaro M., Betta E., Biasioli F., Gasperi F. (2017). Sweet taste in apple: The role of sorbitol, individual sugars, organic acids and volatile compounds. Sci. Rep..

[23] Rymenants M., van de Weg E., Auwerkerken A., De Wit I., Czech A., Nijland B., Heuven H., De Storme N., Keulemans W. (2020). Detection of QTL for apple fruit acidity and sweetness using sensorial evaluation in multiple pedigreed full-sib families. Tree Genet. Genomes.

[24] Morimoto T., Yonemushi K., Ohnishi H., Banno K. (2014). Genetic and physical mapping of QTLs for fruit juice browning and fruit acidity on linkage group 16 in apple. Tree Genet. Mol. Breed..

[25] Maliepaard C., Alston F.H., van Arkel G., Brown L.M., Chevreau E., Dunemann F., Evans K.M., Gardiner S., Guilford P., van Heusden A.W. (1998). Aligning male and female linkage maps of apple (Malus pumila Mill.) using multi-allelic markers. Theor. Appl. Genet..

[26] Joshi A.P.K., Rupasinghe H.P.V., Pitts N.L., Khanizadeh S. (2007). Biochemical characterization of enzymatic browning in selected apple genotypes. Can. J. Plant Sci..

[27] Nicolas J.J., Richard-Forget F.C., Goupy P.M., Amiot M.J., Aubert S.Y. (1994). Enzymatic browning reactions in apple and apple products. Crit. Rev. Food Sci. Nutr..

[28] Fenech M., Amaya I., Valpuesta V., Botella M.A. (2018). Vitamin C content in fruits: Biosynthesis and regulation. Front. Plant Sci..

[29] Kritziger E.C., Bauer F.F., du Toit W.J. (2013). Role of glutathione in winemaking: A review. J. Agric. Food Chem..

[30] Boss P.K., Gardner R.C., Janssen B.-J., Ross G.S. (1995). An apple polypgenol oxidase cDNA is up-regulated in wounded tissues. Plant Mol. Biol..

[31] Janovitz-Klapp A.H., Richard F.C., Goupy M., Nicolas J.J. (1990). Kinetic studies on apple polyphenol oxidase. J. Agric. Food Chem..

[32] Dugé de Bernonville T., Guyot S., Paulin J.-P., Gaucher M., Loufrani L., Henrion D., Derbré S., Guilet D., Richomme P., Dat J.F. (2010). Dihydrochalcones: Implication in resistance to oxidative stress and bioactivities against advanced glycation end-products and vasoconstriction. Phytochemistry.

[33] Yuri A.J., Neira A., Quilodran A., Razmilic I., Motomura Y., Torres C., Palomo I. (2010). Sunburn on apples is associated with increases in phenolic compounds and antioxidant activity as a function of the cultivar and areas of the fruit. J. Food Agric. Environ..

[34] Łata B., Tomala K. (2007). Apple Peel as a Contributor to whole fruit quantity of potentially healthful bioactive compounds. cultivar and year Implication. J Agric. Food Chem..

[35] Li D., Wang P., Luo Y., Zhao M., Chen F. (2017). Health benefits of anthocyanins and molecular mechanisms: Update from recent decade. Crit. Rev. Food Sci. Nutr..

[36] Gosch C., Halbwirth H., Stich K. (2010). Phloridzin: Biosynthesis, distribution and physiological relevance in plants. Phytochemistry.

[37] Mikulic-Petkovsek M., Stampar F., Veberic R. (2007). Parameters of inner quality of the apple scab resistant and susceptible apple cultivars (Malus domestica Borkh.). Sci. Hortic..

[38] Vrhovsek U., Masuero D., Gasperotti M., Franceschi P., Caputi L., Viola R., Mattivi F. (2012). A versatile targeted metabolomics method for the rapid quantification of multiple classes of phenolics in fruits and beverages. J. Agric. Food Chem..

[39] Vanzo A., Janeš L., Požgan F., Bolta Š.V., Sivilotti P., Lisjak K. (2017). UHPLC-MS/MS determination of varietal thiol precursors in Sauvignon Blanc grapes. Sci. Rep..

[40] Zupan A., Mikulic-Petkovsek M., Slantnar A., Stampar F., Veberic R. (2014). Individual phenolic response and peroxidase activity in peel of differently sun-exposed apples in the period favorable for sunburn occurrence. J. Plant Physiol..

[41] Cebulj A., Halbwirth H., Mikulic-Petkovsek M., Veberič R., Slatnar A. (2020). The impact of scald development on phenylpropanoid metabolism based on phenol content, enzyme activity, and gene expression analysis. Hortic. Environ. Biotechnol..

