# Supplementary material for: Apple (Malus domestica Borkh.) Cultivar ‘Majda’, a Naturally Non-Browning Cultivar: An Assessment of Its Qualities
Source: Plants (Basel). 2021 Jul 9;10(7):1402. doi: 10.3390/plants10071402 (PMC8309218; doi:10.3390/plants10071402)
Supplement: Supplementary file 1 [file plants-10-01402-s001.zip › plants-1289870-SI.pdf]

Table S1. The contrast analysis for total sugars with estimated differences between averages and corresponding 95 % confidence intervals (lwr, lower boundary; upr, upper boundary), for time (harvest, storage), location (L1 and L2) and cultivar (GD, 'Golden Delicious'; MA, 'Majda').

Linear hypotheses	Differences of means	lwr	upr
storage GD: L1 / L2	-10.9	-21.7	-0.1
harvest L1:GD / MA	9.1	-1.7	19.9
harvest L2:GD / MA	13.6	2.8	24.4
L1 MA:harvest / storage	-11.4	-22.2	-0.5
L2 MA:harvest / storage	-15.3	-26.1	-4.4

Table S2. The contrast analysis for total organic acids with estimated differences between averages and corresponding 95 % confidence intervals (lwr, lower boundary; upr, upper boundary), for time (harvest, storage), location (L1 and L2) and cultivar (GD, 'Golden Delicious'; MA, 'Majda').

Linear hypotheses	Differences of means	lwr	upr
storage MA:L1 / L2	2.4	0.8	4.0
harvest L1: GD / MA	-2.8	-4.4	-1.2
harvest L2: GD / MA	-2.9	-4.5	-1.3
storage L1: GD / MA	-4.9	-6.6	-3.3
L1 GD:harvest / storage	2.8	1.2	4.4
L2 GD:harvest / storage	2.5	0.9	4.1
L2 MA:harvest / storage	4.2	2.6	5.9

Table S3. The contrast analysis for pH with estimated differences between averages and corresponding 95 % confidence intervals (lwr, lower boundary; upr, upper boundary), for time (harvest, storage), location (L1 and L2) and cultivar (GD, 'Golden Delicious'; MA, 'Majda').

Linear hypotheses	Differences of means	lwr	upr
harvest L1: GD / MA	0.4	0.3	0.5
harvest L2: GD / MA	0.3	0.2	0.4
storage L1: GD / MA	0.5	0.4	0.6
storage L2: GD / MA	0.4	0.3	0.5
L1 GD:harvest / storage	-0.2	-0.2	0.0
L2 GD:harvest / storage	-0.2	-0.3	-0.1

Table S4. The contrast analysis for change in color ($\Delta E\ \Delta t^{-1}$) with estimated ratios of averages and corresponding 95 % confidence intervals (lwr, lower boundary; upr, upper boundary), for time (harvest, storage), location (L1 and L2) and cultivar (GD, 'Golden Delicious'; MA, 'Majda').

Linear hypotheses	Ratio of means	lwr	upr
harvest GD:halves/pomace	0.24	0.14	0.43
harvest MA:halves/pomace	0.23	0.13	0.41
storage GD:halves/pomace	0.49	0.28	0.86
harvest, halves: GD/MA	3.57	2.02	6.33
harvest, pomace: GD/MA	3.41	1.93	6.04
storage, halves: GD/MA	2.15	1.22	3.81
storage, pomace: GD/MA	2.77	1.57	4.91
pomace GD:harvest/storage	1.83	1.03	3.23
halves MA:harvest/storage	0.54	0.31	0.96

Table S5. The contrast analysis for vitamin C, methionine, cysteine and glutathione (GSH and GSSG) with estimated ratios of averages and corresponding 95 % confidence intervals (lwr, lower boundary; upr, upper boundary), for time (harvest, storage), location (L1 and L2) and cultivar (GD, 'Golden Delicious'; MA, 'Majda').

Linear hypotheses	Ratio of means	lwr	upr
Vitamin C			
harvest GD:L1/L2	0.61	0.42	0.9
storage L1: GD/MA	0.24	0.35	0.35
storage L2: GD/MA	0.39	0.57	0.57
L1 GD:harvest/storage	2.71	3.98	3.98
L2 GD:harvest/storage	3.33	4.89	4.89
Methionine			
storage GD:L1/L2	3.18	1.61	6.27
storage MA:L1/L3	2.39	1.21	4.71
storage L1: GD/MA	0.18	0.09	0.36
storage L2: GD/MA	0.14	0.07	0.27
L1 GD:harvest/storage	5.86	2.98	11.55
L2 GD:harvest/storage	23.05	11.7	45.41
Cysteine			
harvest L2: GD/MA	1.25	1.03	1.53
L1 GD:harvest/storage	1.37	1.13	1.68
L2 GD:harvest/storage	1.51	1.24	1.84
GSH			
harvest GD:L1/L2	0.72	0.55	0.94
harvest L1: GD/MA	0.5	0.38	0.66
harvest L1: GD/MA	0.6	0.46	0.78
storage L1: GD/MA	0.52	0.4	0.68
storage L2: GD/MA	0.42	0.32	0.54
L1 GD:harvest/storage	0.7	0.54	0.92
L1 MA:harvest/storage	0.73	0.56	0.95
GSSG			
GD/MA	0.69	0.56	0.85

Table S6. Individual phenols (mean ± SE) in flesh (mg kg^{-1} FW), peels (mg kg^{-1} FW) and leaves (mg kg^{-1} DW). Hydroxycinnamic acids: cryptochlorogenic acid, 4-CQA; chlorogenic acid, 3-CQA; neochlorogenic acid, 5-CQA and p-coumaric acid, p_coum_ac and; Dihydrochalcones: phloridzin, Phl; phloretin 2'-O- xylosyl-glucoside, Phl_xy_phl; 3-hydroxyphloridzin, 3_Hy_phl and 3-hydroxyphloretin, 3_Hyd_phl; flavonols: quercetin-3-rhamnoside, Q-Rha; quercetin-3-rutinoside, Q-Rut and quercetin-3-glycoside + quercetin-3-galactoside, Q-Glc_Gal; flavan-3-ols: catechin, Cat; epicatechin, Epicat; procyanidin B1, P_B1; procyanidin B2 + B4, P_B2_B4.

			Hydroxycinnamic acids								Dihydrochalcones							
**Flesh**			4-CQA		3-CQA		5-CQA		p_coum ac		Phl		Phl_Xy_Glc		3_Hy_phl		3_Hyd_phl	
Cultivar	Location	Time	mean	se	mean	se	mean	se	mean	se	mean	se	mean	se	mean	se	mean	se
'Golden Delicious'	L1	harvest	0.6	0.0	35.2	5.4	0.1	0.0	0.6	0.1	1.4	0.1	0.9	0.2	0.5	0.5	0.0	0.0
		storage	0.7	0.1	68.7	5.7	0.1	0.0	1.0	0.0	4.0	1.1	3.8	0.6	0.9	0.6	0.0	0.0
	L2	harvest	0.6	0.0	56.2	4.6	0.1	0.0	1.0	0.1	1.2	0.2	1.0	0.3	0.4	0.4	0.0	0.0
		storage	0.7	0.1	92.1	9.3	0.1	0.0	1.6	0.1	2.7	0.2	3.4	0.4	1.0	0.6	0.0	0.0
'Majda'	L1	harvest	0.0	0.0	4.9	0.9	0.0	0.0	0.0	0.0	1.8	0.1	0.8	0.2	0.0	0.0	0.0	0.0
		storage	0.2	0.1	5.1	0.2	0.03	0.03	LOQ		1.6	0.2	1.4	0.2	0.0	0.0	0.03	0.03
	L2	harvest	0.0	0.0	4.9	0.7	0.0	0.0	0.0	0.0	1.8	0.3	0.5	0.1	0.0	0.0	0.02	0.02
		storage	0.0	0.0	8.2	1.6	0.05	0.04	LOQ		2.7	0.7	2.3	0.6	0.0	0.0	0.05	0.03

**Peel**		4-CQA2		3-CQA		5-CQA		p_coum ac		Phl		Phl_Xy_Glc		3_Hy_phl		3_Hyd_phl	
Cultivar	Location	mean	se	mean	se	mean	se	mean	se	mean	se	mean	se	mean	se	mean	se
'Golden Delicious'	L1	0.5	0.2	25.0	2.5	0.1	0.0	3.0	0.2	19.3	1.5	12.2	0.8	0.0	0.0	1.2	0.1
	L2	0.6	0.0	41.2	2.9	0.1	0.0	5.5	0.2	17.6	2.3	13.2	0.8	0.0	0.0	0.3	0.1
'Majda'	L1	0.0	0.0	1.4	0.1	0.0	0.0	1.1	0.2	29.3	2.9	5.9	0.5	0.0	0.0	0.6	0.3
	L2	0.0	0.0	1.3	0.1	0.0	0.0	1.9	0.2	25.6	2.1	6.5	0.8	0.0	0.0	1.1	0.5

**Leaves**		Arb		4-CQA2		3-CQA		5-CQA		Phl		Phl_Xy_Glc		3_Hy_phl		3_Hyd_phl	
Cultivar	Location	mean	se	mean	se	mean	se	mean	se	mean	se	mean	se	mean	se	mean	se
'Golden Delicious'	L1	12.4	0.7	1.7	0.2	215.1	21.8	7.2	0.2	5229.5	45.1	29.3	4.2	508.4	30.6	202.6	24.2
	L2	14.7	0.8	1.8	0.3	302.8	24.8	8.4	0.3	5407.3	51.3	15.2	1.1	627.1	75.0	331.5	23.3
'Majda'	L1	9.5	1.5	2.1	0.3	347.5	54.1	3.3	0.5	5148.9	200.7	407.7	39.0	314.4	65.3	707.5	246.4
	L2	13.4	0.6	2.6	0.5	451.5	102.7	4.7	0.6	5779.7	152.5	377.2	15.4	552.0	57.5	201.0	11.1

			Flavonols						Flavan-3-ols							
**Flesh**			Q-Rha		Q-Glc_Gal		Q-rut		Cat		Epicat		P_B1		P_B2_B4	
Cultivar	Location	Time	mean	se	mean	se	mean	se	mean	se	mean	se	mean	se	mean	se
'Golden	L1	harvest	0.1	0.1	0.0	0.0	0.0	0.0	1.4	0.4	3.9	1.0	0.9	0.1	5.0	1.5
Delicious'		storage	0.2	0.0	0.0	0.0	0.0	0.0	3.6	0.5	5.3	0.6	1.7	0.2	14.1	1.7
	L2	harvest	0.3	0.1	0.0	0.0	0.0	0.0	3.0	0.3	5.7	0.4	1.6	0.3	8.5	2.2
		storage	0.4	0.1	0.0	0.0	0.0	0.0	5.1	0.5	10.3	0.6	2.3	0.6	29.0	1.4
'Majda'	L1	harvest	0.3	0.1	0.2	0.0	0.0	0.0	0.0	0.0	0.2	0.1	0.0	0.0	0.0	0.0
		storage	0.3	0.0	0.2	0.0	0.0	0.0	0.2	0.1	0.1	0.0	0.1	0.1	0.4	0.2
	L2	harvest	0.3	0.1	0.1	0.0	0.0	0.0	0.0	0.0	0.2	0.1	0.0	0.0	0.2	0.2
		storage	0.3	0.0	0.2	0.0	0.0	0.0	LOQ		0.0	0.0	0.0	0.0	0.5	0.3

**Peel**		Q-Rha		Q-Glc_Gal		Q-rut		Cat		Epicat		P_B1		P_B2_B4	
Cultivar	Location	mean	se	mean	se	mean	se	mean	se	mean	se	mean	se	mean	se
'Golden	L1	13.9	1.2	42.7	4.3	5.1	1.0	7.8	1.3	26.8	2.0	1.7	0.6	75.1	4.0
delicious'	L2	14.3	2.3	34.8	6.0	2.0	0.8	15.1	0.6	47.5	1.6	6.1	0.8	137.7	7.8
'Majda'	L1	9.3	1.1	64.7	7.2	0.0	0.0	4.4	0.6	7.7	0.8	1.2	0.5	18.6	2.9
	L2	7.3	1.0	38.6	5.4	0.0	0.0	4.3	0.4	14.4	0.8	1.8	0.4	25.4	3.6

**Leaves**		Q-Rha		Q-Glc_Gal		Q-rut		Cat		Epicat		P_B1		P_B2_B4	
Cultivar	Location	mean	se	mean	se	mean	se	mean	se	mean	se	mean	se	mean	se
'Golden	L1	396.4	8.4	711.2	22.2	224.4	22.0	6.1	0.5	99.9	5.9	4.6	0.8	74.3	7.3
delicious'	L2	341.3	6.6	694.9	18.8	406.6	31.2	9.4	1.2	148.9	8.3	12.3	1.6	153.4	9.9
'Majda'	L1	285.4	11.1	657.7	51.8	7.0	2.2	3.3	0.3	33.1	2.2	1.9	0.6	32.2	2.9
	L2	272.1	10.7	752.5	11.1	2.9	1.0	4.9	0.5	49.3	3.6	2.2	0.7	45.2	5.3

LOQ, limit of quantitation

Table S7. The contrast analysis for phenolic groups dihydrochalcones and flavonols in apple flesh with estimated estimated ratios of averages and corresponding 95 % confidence intervals (lwr, lower boundary; upr, upper boundary), for time (harvest, storage), location (L1 and L2) and cultivar (GD, 'Golden Delicious'; MA, 'Majda').

Linear hypotheses	Ratio of means	lwr	upr
Dihydrochalcones			
harvest.GD / storage.GD	0.35	0.23	0.52
harvest.MA / storage.GD	0.32	0.21	0.48
harvest.MA / storage.MA	0.66	0.43	0.99
storage.GD / storage.MA	2.09	1.38	3.15
Flavonols			
GD.L1 - GD.L2	0.41	0.22	0.76
GD.L1 - MA.L1	0.32	0.17	0.59
GD.L1 - MA.L2	0.31	0.17	0.57

Table S8. HSD.test for phenolic groups hydroxycinnamic acids, dihydrochalcones, flavonols and flavan-3-ols in apple flesh, peel and leaves with estimated ratios of averages and corresponding 95 % confidence intervals (lwr, lower boundary; upr, upper boundary), for time (harvest, storage), location (L1 and L2) and cultivar (GD, 'Golden Delicious'; MA, 'Majda').

Flesh - HSD.test	Ratio of means	lwr	upr
Hydroxycinnamic acids			
Harvest / storage	0.64	0.53	0.77
GD / MA	10.98	9.06	13.32
L1 / L2	0.75	0.62	0.91
Flavan-3-ols			
GD / MA	287.14	55.99	1472.66
Peel - HSD.test			
Hydroxycinnamic acids			
GD / MA	12.93	10.77	15.51
L1 / L2	0.67	0.56	0.81
Flavonols			
L1 / L2	1.43	1.05	1.95
Flavan-3-ols			
GD / MA	4.01	3.38	4.77
L1 / L2	0.61	0.51	0.72
Leaves - HSD.test			
Arbutin			
GD / MA	2.13	0.15	4.1
L1 / L2	-3.16	-5.13	-1.18
Hydroxycinnamic acids			
GD / MA	0.7	0.52	0.93
Dihydrochalcones			
GD / MA	0.92	0.88	0.96
L1 / L2	0.94	0.9	0.99
Flavonols			
GD / MA	1.41	1.29	1.53
Flavan-3-ols			
GD / MA	2.89	2.53	3.31
L1 - L2	0.63	0.55	0.72

Table S9. The contrast analysis for PPO and POX with estimated estimated ratios of averages and corresponding 95 % confidence intervals (lwr, lower boundary; upr, upper boundary), for time (harvest, storage), location (L1 and L2) and cultivar (GD, 'Golden Delicious'; MA, 'Majda').

Linear hypotheses	Ratio of means	lwr	upr
PPO			
storage MA: L1/L2	2.00	1.18	3.30
L2 GD:harvest/storage	1.81	1.08	3.04
L2 MA:harvest/storage	1.69	1.01	2.84
POX			
L2 GD:harvest/storage	-0.52	-3.51	0.03

Table S10. Meteorological conditions during vegetation period (1. 4. 2019-30. 9. 2021) on locations L1 and L2

Meteorological variable	L1	L2
Total precepitation (mm)	731.2	752.6
Average daily air temperature (°C)	16.3	17.1
Average minimum daily air temperature (°C)	10.8	11.5
Average maximum daily air temperature (°C)	21.8	23.5
Total solar global radiation in September (W/m^2)	6623	7522

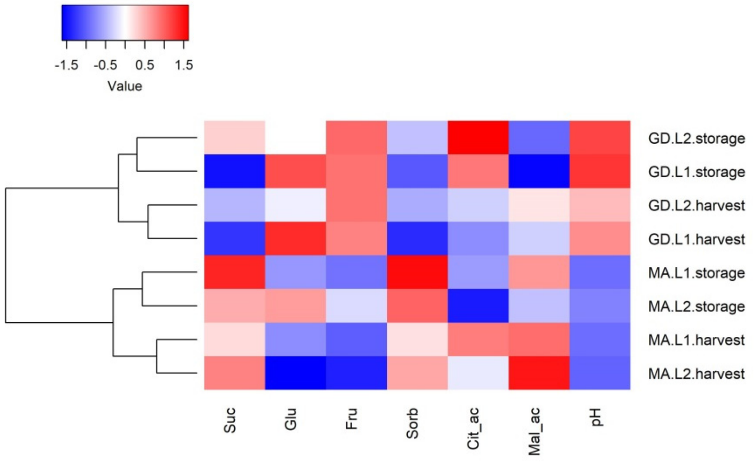

Figure S1. Heatmap for standardized variables: individual sugars: sucrose, Suc; glucose, Glu; fructose, Fru; sorbitol, Sorb; organic acids: citric acid, Cit_ac; malic acid, Mal_ac and pH for cultivars 'Golden Delicious' (GD) and 'Majda' (MA) at two different locations (L1 and L2) at harvest and following storage, dendrogram based on Ward's clustering squared Euclidian distance.

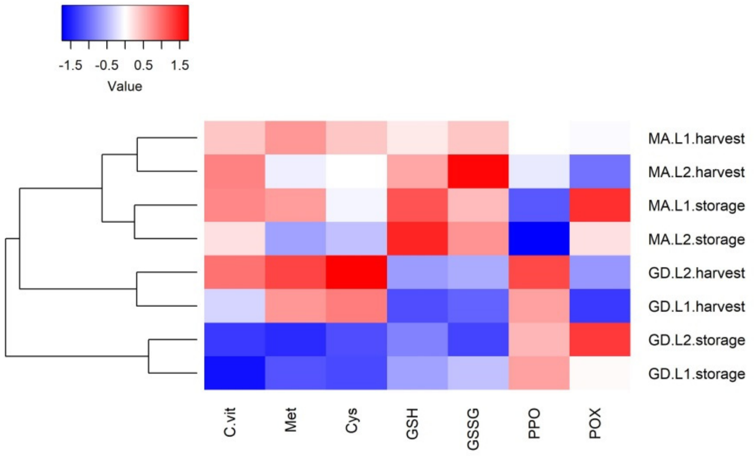

Figure S2. Heatmap for standardized variables: vitamin C, C vit; methionine, Met; cysteine, Cys; reduced glutathione, GSH; oxidised glutathione, GSSG; polyphenol oxidase, PPO; peroxidase, POX for cultivars 'Golden Delicious' (GD) and 'Majda' (MA) at two different locations (L1 and L2) at harvest and following storage, dendrogram based on Ward's clustering squared Euclidian distance.

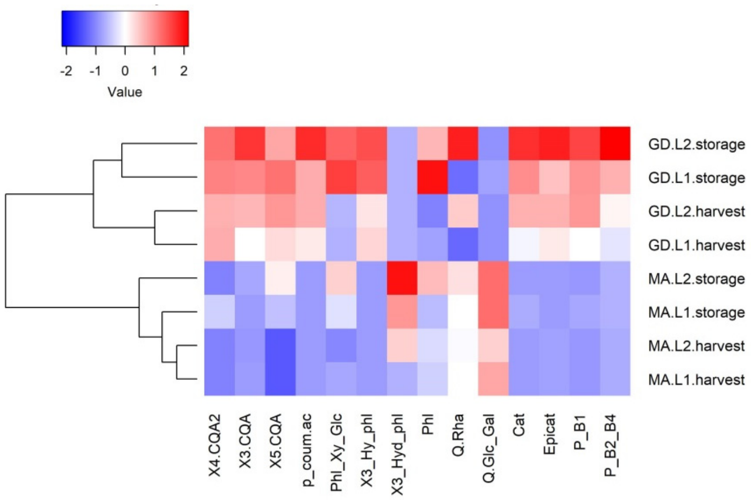

Figure S3. Heatmap for standardized variables: Hydroxycinnamic acids: cryptochlorogenic acid, X4-CQA; chlorogenic acid, X3-CQA; neochlorogenic acid, X5-CQA and p-coumaric acid, p_coum_ac and; Dihydrochalcones: phloridzin, Phl; phloretin 2'-O- xylosyl-glucoside, Phl_xy_phl; 3-hydroxyphloridzin, X3_Hy_phl and 3-hydroxyphloretin, X3_Hyd_phl; flavonols: quercetin-3-rhamnoside, Q-Rha; quercetin-3-rutinoside, Q-Rut and quercetin-3-glycoside + quercetin-3-galactoside, Q-Glc_Gal; flavan-3-ols: catechin, Cat; epicatechin, Epicat; procyanidin B1, P_B1; procyanidin B2 + B4, P_B2_B4 for cultivars 'Golden Delicious' (GD) and 'Majda' (MA) at two different locations (L1 and L2) at harvest and following storage,dendrogram based on Ward's clustering squared Euclidian distance.

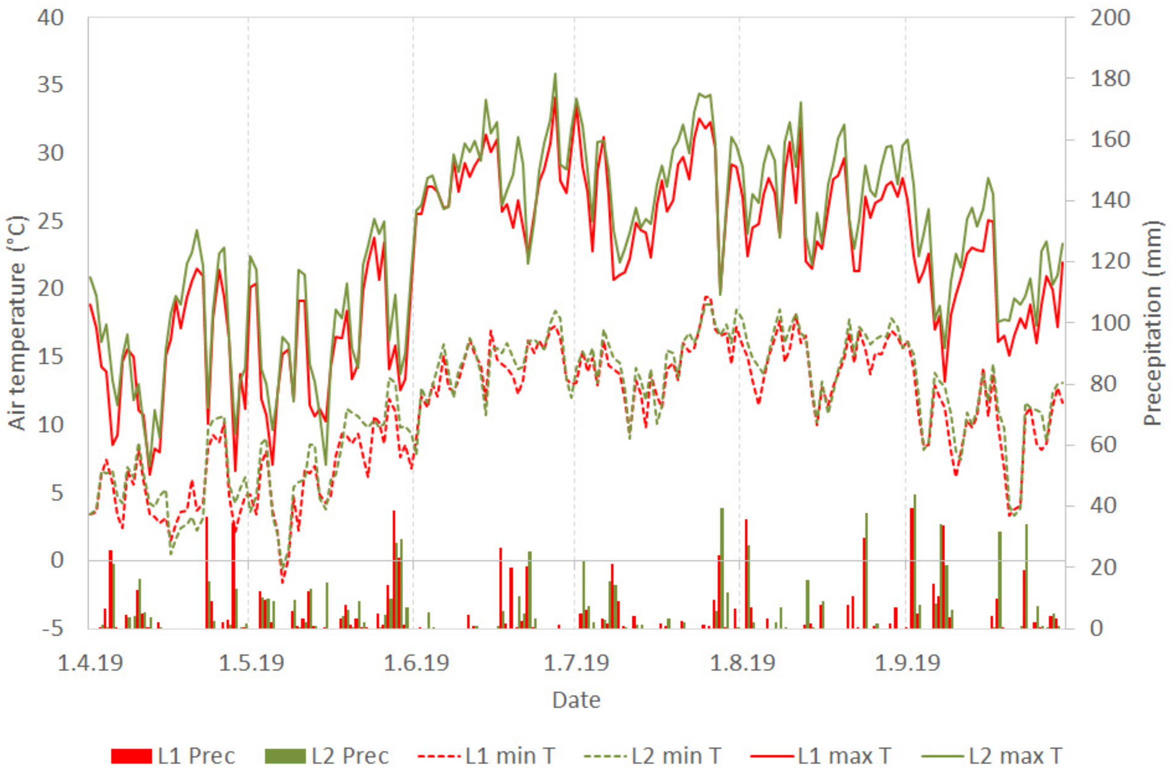

Figure S4. Daily precipitations, daily minimum and maximum air temperature for the locations L1 and L2
